# Do electronic cigarettes increase cigarette smoking in UK adolescents? Evidence from a 12-month prospective study

**DOI:** 10.1136/tobaccocontrol-2016-053539

**Published:** 2017-08-17

**Authors:** Mark Conner, Sarah Grogan, Ruth Simms-Ellis, Keira Flett, Bianca Sykes-Muskett, Lisa Cowap, Rebecca Lawton, Christopher J Armitage, David Meads, Carole Torgerson, Robert West, Kamran Siddiqi

**Affiliations:** 1 School of Psychology, University of Leeds, Leeds, UK; 2 Department of Psychology, Manchester Metropolitan University, Manchester, UK; 3 Centre for Health Psychology, The Science Centre, Staffordshire University, Stoke-on-Trent, UK; 4 Division of Psychology and Mental Health, Manchester Centre for Health Psychology, Manchester Academic Health Science Centre, University of Manchester, Manchester, UK; 5 Institute of Health Sciences, University of Leeds, Leeds, UK; 6 School of Education, Durham University, Durham, UK; 7 Department of Health Sciences, University of York, York, UK

**Keywords:** electronic nicotine delivery systems, e-cigarettes, smoking, harm reduction, prevention

## Abstract

**Background:**

In cross-sectional surveys, increasing numbers of adolescents report using both electronic cigarettes (e-cigarettes) and cigarettes. This study assessed whether adolescent e-cigarette use was associated prospectively with initiation or escalation of cigarette use.

**Methods:**

Data were from 2836 adolescents (aged 13–14 years at baseline) in 20 schools in England. At baseline, breath carbon monoxide levels, self-reported e-cigarette and cigarette use, sex, age, friends and family smoking, beliefs about cigarette use and percentage receiving free school meals (measure of socioeconomic status) were assessed. At 12-month follow-up, self-reported cigarette use was assessed and validated by breath carbon monoxide levels.

**Results:**

At baseline, 34.2% of adolescents reported ever using e-cigarettes (16.0% used only e-cigarettes). Baseline ever use of e-cigarettes was strongly associated with subsequent initiation (n=1726; OR 5.38, 95% CI 4.02 to 7.22; controlling for covariates, OR 4.06, 95% CI 2.94 to 5.60) and escalation (n=318; OR 1.91, 95% CI 1.14 to 3.21; controlling for covariates, this effect became non-significant, OR 1.39, 95% CI 0.97 to 1.82) of cigarette use.

**Conclusions:**

This is the first study to report prospective relationships between ever use of e-cigarettes and initiation and escalation of cigarette use among UK adolescents. Ever use of e-cigarettes was robustly associated with initiation but more modestly related to escalation of cigarette use. Further research with longer follow-up in a broader age range of adolescents is required.

## Introduction

Electronic cigarettes (e-cigarettes) deliver inhaled aerosol usually containing nicotine. E-cigarettes are thought to have minimal impact on morbidity and mortality^1 2^ and are recognised as harm reducing for adult smokers.^2–4^ Although rates of adolescent regular use of e-cigarettes are low, rates of ever use are substantial (13%–22%) and have increased over recent years, whereas rates of cigarette use have decreased over the same period both in the USA^5–7^ and UK.^8–15^ Nevertheless, the possible relationship between adolescent e-cigarette use and the initiation and escalation of cigarette use remains under-researched.

Longitudinal data on e-cigarette use and subsequent cigarette use are currently limited to US samples based on unverified self-reported measures.^16–19^ For example, two US studies reported baseline e-cigarette use to be positively associated with the initiation of cigarette use 12 months later in 14-year olds controlling for various predictors of smoking (OR 1.75, 95% CI 1.10 to 2.77; OR 2.87, 95% CI 2.03 to 4.05).^17 18^ Barrington-Trimis *et al* 16 reported similar findings over 16 months in 17-year-olds (OR 6.17, 95% CI 3.30 to 11.6), whereas Wills *et al* 19 reported that e-cigarette use was linked to initiation (OR 2.87, 95% CI 2.03 to 4.05) but not to escalation of smoking over 12 months in a sample of adolescents aged 14–15 years.

This study is novel in assessing these relationships between e-cigarette use and subsequent cigarette use in a sample of UK adolescents and in exploring a number of previously unexamined smoking risk factors as covariates and moderators. In particular, we investigated the extent to which baseline ever use of e-cigarettes was associated with the initiation or escalation of cigarette use (objectively validated) 12 months later in a sample of UK adolescents aged 13–14 years. The impact of controlling for various smoking risk factors such as friends and family smoking and their moderating effects was also explored.

## Methods

### Participants and procedures

Data were collected as part of a 4-year cluster randomised controlled trial of a school-based smoking initiation intervention^20 21^ based on implementation intentions.^22^ Data from 2836 adolescents (13–14 years at baseline) in the 20 control schools are reported here. Head teachers consented to school participation with parents given the option to withdraw children from the study. Adolescents consented by completing questionnaires matched across time points using a personally generated code. The data reported here are from waves 3 (September–December 2014; referred to as *baseline*) and 4 (September–December 2015; referred to as *follow-up*) of the trial when e-cigarette use measures were added to the data collection.

The Faculty of Medicine, University of Leeds, UK, ethical review committee approved the study (reference 12–0155).

### Measures

Cigarette use was assessed using a standardised measure^23^ at both time points; adolescents ticked one of the following: ‘I have never smoked; I have only tried smoking once; I used to smoke sometimes, but I never smoke cigarettes now; I sometimes smoke cigarettes now, but I don’t smoke as many as one a week; I usually smoke between one and six cigarettes a week; and I usually smoke more than six cigarettes a week’. Self-reported smoking was validated against a measure of breath carbon monoxide (CO) levels (using Micro+ Smokerlyzer CO Monitor; Bedfont Scientific Limited, Kent, England, UK). Such measures are reliable and valid ways of assessing regular cigarette smoking^24 25^ but not occasional smoking due to the short half-life (4–6 hours) of breath CO.

E-cigarettes/vapourisers were described as ‘a tube that sometimes looks like a normal cigarette and has a glowing tip. They all puff a vapour that looks like smoke but unlike normal cigarettes, they don’t burn tobacco’. Awareness (‘Have you ever heard of e-cigarettes or vapourisers?’ yes I have; no I haven’t; I don’t know) and use (‘Which ONE of the following is closest to describing your experience of e-cigarettes or vapourisers?’ I have never used them; I have tried them once or twice; I use them sometimes (more than once a month but less than once a week); I use them often (more than once a week)) of e-cigarettes were tapped by single items.

Other measures were assessed as covariates/moderators. Percentage of children at a school eligible for free school meals was used as an indicator of socioeconomic status.^26^ Sex and age were measured (age not used in analyses as adolescents from one school year). Family smoking was assessed using the question, ‘Who smokes in your family now? Tick all the people who smoke at the moment’, followed by a list of family members (zero to nine family members marked; scored as 0, 1, 2 or 3 or more). Friends’ smoking was assessed using the question, ‘How many of your friends smoke?’ none of them; only a few; half and half; most but not all; all of them (scored as none of them, a few or most (last three categories)).

Baseline health cognitions about smoking^21^ were assessed as mean of multiple items on five-point scales (high scores indicated negative views of smoking): intention was tapped by three statements (‘I plan not to smoke’, ‘I don’t want to smoke’ and ‘I will try not to smoke’; strongly disagree to strongly agree; Cronbach’s alpha 0.90), attitude by seven statements (‘For me, smoking would be… good–bad; beneficial–harmful; pleasant–unpleasant; enjoyable–unenjoyable; wise–foolish; fun–not fun; healthy–unhealthy’; Cronbach’s alpha 0.87), norms by five statements (‘Most of my friends think…’; ‘My best male friend thinks…’; ‘My best female friend thinks…’; ‘My family think…’; ‘People who are important to me think…’; I should smoke–I should not smoke; Cronbach’s alpha 0.79), perceived behavioural control by three statements (‘I am confident I could resist smoking’, strongly disagree to strongly agree; ‘For me to not smoke would be…’, difficult–easy; ‘How much control do you feel you have over not smoking?’ no control–complete control; Cronbach’s alpha 0.69) and self-efficacy by six statements (‘I can say no to smoking, even at school’; ‘I can say no to smoking even when I am offered a cigarette’; ‘I can say no to smoking, even if my friends want me to smoke’; ‘I can say no to smoking, even if I was the only one in the group not smoking’; ‘I can say no to smoking, even if I feel a bit left out of the group’; ‘I can say no to smoking, even if I feel like smoking’; strongly disagree-strongly agree; Cronbach’s alpha 0.91).

### Data analysis

We tested for differences on each baseline measure between adolescents who had complete versus missing values on one or more measures using χ^2^ tests and t-tests. Among respondents completing all measures, we report descriptives on baseline measures for three subsamples: full cross-sectional sample, longitudinal subsample of baseline never users of cigarettes and longitudinal subsample of baseline occasional users of cigarettes. The relationship between e-cigarette and cigarette use was examined next in the same three subsamples. Self-rated smoking was validated against breath CO levels at baseline and follow-up using Games–Howell post hoc tests based on 1000 bootstrapped resamples because the data were skewed and had unequal variances.

Given the problems with imputing values for outcome variables,^27^ attrition analyses were used to assess biases in all baseline measures in those with and without matched follow-up data (at follow-up 1=data missing; 0=data available) in the two longitudinal subsamples using multilevel logistic regressions (in R) to assess model fit (Akaike Information Criterion) and, for each predictor, the odds ratios (OR), 95% CIs and p value. The main analyses used the same analysis to predict follow-up initiation (1=smoked; 0=never smoked) or escalation (0=never, once or used to smoke cigarettes; 1=rarely, occasional or frequent cigarette smoking) of smoking based on ever use of e-cigarettes and covariates. E-cigarette use was dichotomised into never versus ever use due to few regular users. Model 1 controlled for the clustering of adolescents within schools, and baseline e-cigarette ever use was a predictor; model 2 added baseline covariates; and model 3 tested interactions between each covariate and e-cigarettes ever use. To assess the impact of baseline missing values, we repeated the regressions with imputation.^28^

## Results

### Sample description

At baseline, full data were available on 2836 adolescents, who did not differ (p>0.05) from those with missing data (N=58–92) on all measures except sex (p=0.001; boys less likely to have complete data) and norms (p=0.02; those with lower norms to not smoke less likely to have complete data).

Table 1 provides descriptive data on baseline measures for respondents who completed all measures. The cross-sectional sample (table 1) was mostly aged 13 years, approximately half boys, and a majority not having ever used e-cigarettes or cigarettes. Levels of e-cigarette awareness and use were lower in the never smoking subsample (table 1: 80.0% heard of, 19.9% used e-cigarettes) compared with the subsample reporting occasional smoking (table 1: 90.0% heard of, 78.0% used e-cigarettes).

**Table 1:** Descriptive data for the full sample and subsamples

		Cross-sectional sample (total N=2836)		Longitudinal sample of baseline never used cigarettes (total n=1726)		Longitudinal sample of baseline once/ used to use cigarettes (total n=318)	
		N/M	(%/SD)	N/M	(%/SD)	N/M	(%/SD)
Age		13.18	(0.39)	13.18	(0.39)	13.17	(0.39)
Sex	Boy	1411	(49.8%)	898	(48.0%)	164	(51.6%)
	Girl	1425	(50.2%)	898	(52.0%)	154	(48.4%)
Heard of e-cigarettes (baseline)	No	346	(12.2%)	227	(13.2%)	24	(7.5%)
	Yes	2383	(84.2%)	1381	(80.0%)	286	(90.0%)
	Don’t know	103	(3.2%)	118	(6.8%)	8	(2.5%)
Ever used e-cigarettes (baseline)	No	1867	(65.8%)	1383	(80.1%)	70	(22.0%)
	Yes	969	(34.2%)	343	(19.9%)	248	(78.0%)
Ever used cigarettes (baseline)	No	2196	(77.4%)	1726)	(100.0%	0	(0.0%)
	Yes	640	(22.6%)	0	(0.0%)	318	(100.0%)
Family smokers = none		898	(31.7%)	666	(38.6%)	42	(13.2%)
Family smokers = one		852	(30.0%)	534	(30.9%)	88	(27.7%)
Family smokers = two		517	(19.2%)	298	(17.3%)	74	(23.2%)
Family smokers = three or more		569	(20.1%)	228	(13.2%)	114	(35.8%)
Friend smokers = none		1384	(48.8%)	1050	(60.8%)	67	(21.1%)
Friend smokers = a few		1135	(40.0%)	613	(35.5%)	189	(59.4%)
Friend smokers = most		317	(11.2%)	63	(3.7%)	62	(19.5%)
Intentions		4.69	(0.77)	4.87	(0.50)	4.48	(0.76)
Attitude		4.73	(0.57)	4.88	(0.32)	4.51	(0.65)
Perceived norms		4.81	(0.57)	4.91	(0.30)	4.66	(0.50)
Perceived behavioural control		4.61	(0.72)	4.78	(0.49)	4.43	(0.71)
Self-efficacy		4.64	(0.77)	4.83	(0.47)	4.41	(0.82)
Free school meals^*^		14.24	(6.63)	13.82	(6.55)	15.57	(6.35)

*Mean and SD for this variable based on school-level data.

At baseline and follow-up, CO levels were low and not significantly different between those reporting they never smoked, had only tried smoking once, used to smoke sometimes or smoked sometimes but not as many as one per week; CO levels were significantly higher (p<0.05) among those reporting they smoked 1–6 or >6 cigarettes per week but not significantly different across these latter two categories.

### Simple relationships between use of e-cigarettes and cigarettes

Table 2 reports the relationship between e-cigarette and cigarette use in the three subsamples. Table 2A shows the cross-sectional relationship: 61.5% of the sample had tried neither e-cigarettes nor cigarettes, 16.0% had tried e-cigarettes but not cigarettes, 4.4% had tried cigarettes but not e-cigarettes and 18.2% had used both.

**Table 2:** Relationships between cigarette and e-cigarette use: (A) cross-sectional relationships between baseline cigarette and e-cigarette use; (B) prospective relationships between cigarette use at 1-year follow-up and e-cigarette use at baseline among baseline never used cigarettes; (C) prospective relationships between cigarette use at 1-year follow-up and e-cigarette use at baseline among baseline used once or used to use cigarettes

Cigarette Use	Baseline e-cigarette use			
	Never	Tried	Infrequent	Frequent
		(1–2 times)	(1/month–1/week)	(>1/week)
	n (%)	n (%)	n (%)	n (%)
A. Cross-sectional relationships at baseline (n=2836)				
Never	1743 (61.5)	407 (14.4)	40 (1.4)	6 (0.2)
Once	90 (3.2)	201 (7.1)	57 (2.0)	10 (0.4)
Used to	20 (0.7)	59 (2.1)	38 (1.3)	22 (0.8)
Rarely (<1/week)	8 (0.3)	15 (0.5)	31 (1.1)	19 (0.7)
Occasional (1–6/week)	1 (0.0)	6 (0.2)	20 (0.7)	10 (0.4)
Frequent (>6/week)	5 (0.2)	7 (0.2)	6 (0.2)	15 (0.5)
B. Longitudinal relationships for baseline never users of cigarettes (n=1726)				
Never	1259 (72.9)	211 (12.2)	13 (0.8)	1 (0.1)
Once	86 (5.0)	65 (3.8)	8 (0.5)	0 (0.0)
Used to smoke	19 (1.1)	19 (1.1)	1 (0.1)	1 (0.1)
Rarely (<1/week)	11 (0.6)	12 (0.7)	1 (0.1)	0 (0.0)
Occasional (1–6/week)	5 (0.3)	3 (0.2)	2 (0.1)	0 (0.0)
Frequent (>6/week)	3 (0.2)	1 (0.1)	3 (0.2)	2 (0.1)
C. Longitudinal relationships for baseline triers of cigarettes (n=318)				
No change	61 (19.2)	131 (41.2)	43 (13.5)	14 (4.4)
Escalation	9 (2.8)	38 (11.9)	17 (5.3)	5 (1.6)

Table 2B shows the longitudinal relationship between baseline e-cigarette use and follow-up cigarette use in the baseline never smokers; initiation of cigarette use in the next 12 months rose from 9.0% to 34.4%, respectively, in baseline never versus ever used e-cigarettes. Baseline CO levels were low among the self-reported never smokers, and exclusion of adolescents with higher baseline CO levels (>2 ppm) did not substantively change the regression findings. CO levels at follow-up were significantly higher among those classified as initiating compared with not initiating cigarette use (p<0.05).

Table 2C shows the longitudinal relationship between e-cigarette use at baseline and escalation of cigarette use at follow-up among baseline occasional smokers; escalation in the next 12 months rose from 12.9% to 24.2%, respectively, in those never versus ever having used e-cigarettes at baseline. Baseline CO levels were low among those self-reporting that they had only once used or former smokers and exclusion of adolescents with higher baseline CO levels (>2 ppm) did not substantively change the regression findings. CO levels at follow-up were significantly higher among those classified as escalating versus not escalating smoking (p<0.001).

### Attrition analyses

At baseline, 2196 adolescents (77.4%) reported never having smoked but only 1726 adolescents (78.6%) could be matched across time points. The similar number of adolescents completing questions at each time point (total N=2928 and 2747 at baseline and follow-up, respectively) suggests that attrition was principally due to a failure to match personally generated codes.

Analyses (table 3) indicated no significant effects for baseline ever used e-cigarettes, friends’ smoking, attitude, norms, perceived behavioural control, self-efficacy or free school meals on missingness; however, there were significant effects for sex (OR 0.70, 95% CI 0.56 to 0.86; girls less likely to be missing), family smoking (OR 1.53, 95% CI 1.10 to 2.12; with three or more family members who smoked more likely to be missing) and intention (OR 0.77, 95% CI 0.62 to 0.96; with weaker intentions not to smoke more likely to be missing).

**Table 3:** Association of baseline measures with missingness (1=absent) at follow-up for baseline never used cigarettes (n=2196; left-hand column) and baseline once or used to use cigarettes (n=497; right-hand column)

Predictors	Baseline never used cigarettes		Baseline once or used to use cigarettes	
	OR (95% CI)	p Value	OR (95% CI)	p Value
Never used e-cigarettes	1.00		1.00	
Ever used e-cigarettes	1.11 (0.85 to 1.46)	0.43	0.83 (0.51 to 1.35)	0.44
Friend smokers= none	1.00		1.00	
Friend smokers=a few	1.18 (0.93 to 1.49)	0.18	2.08 (1.12 to 3.82)	0.019
Friend smokers= most	1.36 (0.78 to 2.39)	0.28	4.33 (2.10 to 8.95)	<0.001
Male	1.00		1.00	
Female	0.70 (0.56 to 0.86)	<0.001	0.84 (0.6 to 1.26)	0.40
Family smokers = none	1.00		1.00	
Family smokers = one	1.29 (0.99 to 1.67)	0.057	0.90 (0.47 to 1.71)	0.74
Family smokers = two	1.10 (0.79 to 1.51)	0.58	0.97 (0.50 to 1.89)	0.93
Family smokers = three or more	1.53 (1.10 to 2.12)	0.01	0.81 (0.43 to 1.53)	0.51
Intentions	0.77 (0.62 to 0.96)	0.02	0.99 (0.71 to 1.38)	0.95
Attitudes	0.93 (0.65 to 1.31)	0.66	1.29 (0.86 to 1.93)	0.22
Norms	0.95 (0.66 to 1.37)	0.78	0.99 (0.65 to 1.52)	0.97
Perceived behavioural control	0.91 (0.73 to 1.14)	0.42	0.64 (0.46 to 0.88)	0.006
Self-efficacy	1.25 (0.95 to 1.64)	0.11	1.15 (0.79 to 1.67)	0.46
Free school meals	1.03 (0.97 to 1.08)	0.34	1.01 (0.97 to 1.06)	0.49

Baseline never used cigarettes, AIC=2222.6; baseline once or used to use cigarettes, AIC=658.7.

At baseline, 497 adolescents reported trying or past use of cigarettes. We matched 318 adolescents (64.0%) across time points. Analyses indicated no significant effects for baseline ever used e-cigarettes, sex, family smoking, intention, attitude, perceived behavioural control, self-efficacy and free school meals on missingness (table 3); however, there were significant effects for friends’ smoking (OR 2.08, 95% CI 1.12 to 3.82 for few friends smoking; OR 4.33, 95% CI 2.10 to 8.95 for most friends smoking; with a few or most friends who smoked more likely to be missing) and perceived behavioural control (OR 0.64, 95% CI 0.46 to 0.88; with weaker perceived behavioural control over not smoking more likely to be missing).

### Prospective analyses

Initiation of cigarette use at follow-up was predicted by having ever used e-cigarettes at baseline (table 4, model 1; OR 5.38, 95% CI 4.02 to 7.22) and remained so when controlling for covariates (table 4, model 2; OR 4.06, 95% CI 2.94 to 5.60). Initiation of cigarette use was significantly higher in adolescents who at baseline were ever users of e-cigarettes, had either a few or most friends who smoked and had one, two or three or more family members who smoked, but was significantly lower in adolescents with stronger intentions (not to smoke). Exploratory analyses revealed that baseline friends’ smoking was a statistically significant moderator (p<0.001; all other moderators p>0.43). Decomposition of the moderation effect (table 4, model 3) indicated that the the impact of ever used e-cigarettes on likelihood of initiating cigarette use was attenuated among those with a few or most friends who smoked at baseline. Multiple imputation resulted in an additional 28 cases in this analysis. The estimated model coefficients showed very little change (mostly <1%), and there was no change in the interpretation.

**Table 4:** Association of baseline ever used e-cigarettes with ever used cigarettes at follow-up (among never users of cigarettes at baseline; n=1726; left-hand column) or increased use of cigarettes at follow-up (among baseline once or used to use cigarettes; n=318; right-hand column) controlling for clustering by school

Predictors	Baseline never used cigarettes		Baseline once or used to use cigarettes	
	OR (95% CI)	p Value	OR (95% CI)	p Value
Model one without covariates				
Never used e-cigarettes	1.00		1.00	
Ever used e-cigarettes	5.38 (4.02 to 7.22)	<0.001	2.16 (1.01 to 4.62)	0.046
Model two with covariates				
Never used e-cigarettes	1.00		1.00	
Ever used e-cigarettes	4.06 (2.94 to 5.60)	<0.001	1.89 (0.82 to 4.33)	0.13
Friend smokers = none	1.00		1.00	
Friend smokers = a few	1.87 (1.35 to 2.58)	<0.001	1.15 (0.50 to 2.66)	0.75
Friend smokers = most	2.99 (1.52 to 5.87)	0.001	3.23 (1.19 to 8.77)	0.022
Male	1.00		1.00	
Female	1.32 (0.97 to 1.79)	0.08	0.83 (0.45 to 1.52)	0.55
Family smokers = none	1.00		1.00	
Family smokers = one	0.76 (0.51 to 1.13)	0.18	1.69 (0.61 to 4.68)	0.31
Family smokers = two	2.05 (1.37 to 3.06)	<0.001	1.41 (0.48 to 4.12)	0.53
Family smokers = three or more	1.90 (1.23 to 2.94)	0.004	1.23 (0.45 to 3.41)	0.69
Intentions	0.70 (0.52 to 0.96)	0.03	1.50 (0.87 to 2.57)	0.14
Attitudes	0.68 (0.44 to 1.04)	0.08	0.51 (0.28 to 0.90)	0.020
Norms	0.89 (0.57 to 1.39)	0.61	1.12 (0.56 to 2.23)	0.75
Perceived behavioural control	1.00 (0.73 to 1.37)	0.99	0.99 (0.58 to 1.69)	0.96
Self-efficacy	1.09 (0.75 to 1.57)	0.66	0.57 (0.35 to 0.94)	0.027
Free school meals	0.99 (0.97 to 1.02)	0.60	1.01 (0.96 to 1.07)	0.62
Model three with covariates and interactions				
Never used e-cigarettes and Friend smokers = none	1.00			
Ever used e-cigarettes and friend smokers = none	7.74 (4.68—12.79)	<0.001		
Never used e-cigarettes and Friend smokers = a few	2.57 (1.72 to 3.84)	<0.001		
Ever used e-cigarettes and friend smokers = a few	7.84 (5.08–12.09)	<0.001		
Never used e-cigarettes and friend smokers = most	6.32 (2.68 to 14.91)	<0.001		
Ever used e-cigarettes and friend smokers = most	8.75 (3.68–20.83)	<0.001		
Male	1.00			
Female	1.37 (1.01 to 1.86)	0.04		
Family smokers = none	1.00			
Family smokers = one	0.76 (0.51 to 1.14)	0.19		
Family smokers = two	2.02 (1.35 to 3.03)	<0.001		
Family smokers = three or more	1.87 (1.21 to 2.90)	0.005		
Intentions	0.70 (0.52 to 0.96)	0.03		
Attitudes	0.67 (0.44 to 1.01)	0.06		
Norms	0.91 (0.59 to 1.41)	0.69		
Perceived behavioural control	1.00 (0.73 to 1.37)	0.99		
Self-efficacy	1.09 (0.75 to 1.59)	0.65		
Free school meals	0.99 (0.96 to 1.02)	0.47		

Follow-up ever used cigarettes: model without covariates, AIC=1281.3; model with covariates, AIC=1226.5; model with covariates and interactions, AIC=1218.7; follow-up escalation of cigarette use: model without covariates, AIC=334.1; model with covariates, AIC=327.5.

Table 4 also reports the results of the regressions to predict escalation of cigarette use at follow-up. In model 1, ever use of e-cigarettes at baseline was a significant predictor of escalation of cigarette use (OR 2.16, 95% CI 1.01 to 4.62). In model 2, ever use of e-cigarettes at baseline became a non-significant predictor of escalation when controlling for covariates (OR 1.89, 95% CI 0.82 to 4.33). Escalation of cigarette use was significantly higher in adolescents who had most friends who smoked, but was significantly lower in those adolescents with stronger attitudes (not to smoke) and intentions (not to smoke). Exploration of moderation effects revealed that two interactions were statistically significant (attitudes, p=0.01; intentions, p=0.02), although decomposition of these effects did not reveal significant effects of e-cigarette use on escalation of cigarette use at different levels of either moderator (p>0.20). None of the other moderators approached statistical significance (p>0.16). Multiple imputation did not change any values or the analyses.

The ORs based on logistic regression analyses reported in table 4 may overestimate the degree of association between e-cigarette use and subsequent smoking because the prevalence of the outcome exceeds the usual 15% cut-off. To assess the degree of overestimation, we ran the initial models (model 1 in table 4) using a log binomial model. For the analyses of never smokers, the degree of association was reduced but remained statistically significant: incidence relative risk (IRR) was 3.85 (95% CI 3.07 to 4.82), p<0.001. For the analyses of smoking escalation, the degree of association was also reduced and no longer statistically significant: IRR=1.81 (95% CI 095 to 3.44), p=0.071.

## Discussion

We showed that ever use of e-cigarettes is associated with initiation of cigarette use; an effect that remains when controlling for various predictors of smoking. Our study in UK adolescents (13–14 years old) found patterns similar to those reported in longitudinal studies among adolescents aged 13–14 years and older^16–19^ in the USA with comparable sized ORs (the IRR was also of a comparable magnitude). Together, these studies suggest that it is unlikely that the high rates of dual use of e-cigarette and cigarette use observed in the USA^5–7^ and UK^8–15^ in cross-sectional surveys of adolescents are entirely attributable to cigarette users subsequently taking up e-cigarettes. A significant minority of adolescents try e-cigarettes first (19.9% here) and later initiate cigarette use. Our findings also indicated that the association between ever use of e-cigarettes and initiation of cigarette use was particularly strong among adolescents with no friends who smoked, a group usually considered to be less susceptible to smoking initiation (see the study by Barrington-Trimis *et al* 16 for similar moderation effect among those with low intentions to smoke). In relation to escalation of cigarette use, the OR showed that ever use of e-cigarettes is associated with subsequent escalation, although this effect was attenuated when using the IRR or when controlling for covariates. However, given the limited numbers escalating their cigarette use in this study and lack of support in other studies, these findings should be treated cautiously (eg, other studies either did not find e-cigarette use to be related to change in frequency of smoking among baseline ever-smokers,^19^ or found that baseline frequency of use of e-cigarettes was only associated with follow-up smoking frequency among baseline non-smokers and not among baseline infrequent or frequent smokers^29^).

Our research provides limited insights into the mechanism relating ever use of e-cigarettes to subsequent initiation and escalation of cigarette use. In principle, it is possible that e-cigarette use in adolescents is a marker for those who would have initiated or escalated cigarette use even if e-cigarettes had not been available. Among such adolescents, the availability of e-cigarettes may have simply delayed initiation or escalation. However, at least in relation to initiation, the fact that e-cigarette use was a bigger risk factor in groups considered least at risk (ie, no friends who smoke at baseline) argues against this (see the study by Barrington-Trimis *et al* 19 for a similar moderator effect also difficult to reconcile with this explanation). It is also plausible that the use of e-cigarettes might lead to initiation and escalation in cigarette use by normalising any kind of nicotine use, by developing nicotine addiction (if the e-cigarettes contain nicotine) or by developing friendship networks with smokers and decreasing the perceived risks of smoking.^30–32^ However, there is no direct evidence yet to suggest that ever use of e-cigarettes normalises cigarette use.

Given the lack of clarity regarding the mechanism linking e-cigarette and cigarette use, we need to be cautious in making policy recommendations based on our findings. We acknowledge that since our survey, UK legislation has been put in place, including bans on marketing and selling e-cigarettes to minors. UK agencies are required to enforce age of sale, child and tamper proof packaging and display age of sale signage and health warnings on e-cigarette packaging. Nevertheless, our findings emphasise the value of regulating the marketing and sale of e-cigarettes to minors in countries without such measures, particularly given that e-cigarette advertising has been shown to reduce perceived harm of occasional smoking.^33^

Our study’s strengths include a large demographically diverse sample, measurement of e-cigarette and cigarette use over 12 months, exploration of initiation and escalation of cigarette use, validation of smoking measures and exploration of covariates and moderators not previously examined. There are also weaknesses. First, our study had a relatively high attrition. This was principally attributable to problems in matching participants’ personally generated anonymous codes, although attrition analyses indicated relatively modest biases in the final compared with initial sample. Second, like other similar studies, we focused on self-reported e-cigarette and cigarette use. Although we validated the self-reported smoking against an objective measure of CO, we did not have a way of validating e-cigarette use. Third, we failed to distinguish types of e-cigarette use (e-cigarettes vary in a number of ways, including the delivery method and whether they contain nicotine). Furthermore, our description of e-cigarettes and the timing of our survey might have restricted our study to first-generation devices, in which their nicotine delivery profile mimic less closely to cigarettes than do more recent generations.^34^ Exploring relationships between use of new generations of e-cigarettes both containing nicotine or not and subsequent cigarette use is an important issue for further research. The current research focused on cigarette use, although other studies have reported similar effects with various tobacco products.^18^

A fourth limitation concerns our main analyses (table 4), which were restricted to ever use of e-cigarettes, and we were unable to test whether more regular use of e-cigarettes was more strongly associated with initiating or escalating cigarette use (see table 2; see the study by Warner^6^ for cross-sectional data). Relatedly, our analyses of impacts on escalation should be treated cautiously given the limited numbers escalating cigarette use during the period studied and the fact that our findings conflict with published work.^19^ Fifth, our research was restricted to a limited geographical area (two English counties), although it did extend findings from several US states. Sixth, our research focused on a limited age range (baseline: 13–14 years; most published studies^17–19^ are with this age group). Future studies should explore effects in different aged adolescents and over varying time periods. Finally, our research could only consider a finite number of covariates and moderators, and it is plausible that important factors were omitted. Previous related studies^16–19^ have examined various other factors (eg, sensation seeking, impulsivity, other substance use, delinquent behaviour, academic performance and race/ethnicity). It would be valuable to test these additional covariates and moderating variables in future work.

In summary, this is the first study to report longitudinal relationships between ever use of e-cigarettes and initiation or escalation of cigarette use among UK adolescents. Despite measuring and accounting for the influence of a broad range of variables in this and other studies,^16–19^ it is possible that any third variables could have been responsible for the observed relationships. Therefore, while acknowledging that a causal relationship may be plausible, we cannot confirm this based on our findings and the trends observed over the same time period in the UK; rates of e-cigarette use have increased, but the rates of cigarette use have continued to decline. Future research could seek to disentangle these apparently contrary findings and assess dose–response relationships between e-cigarette and cigarette use over longer-time periods in a broader age range of adolescents while controlling for a range of covariates and assessing the impact of antismoking interventions.

**What this paper adds**

**Previous research:** In cross-sectional surveys of UK adolescents, electronic cigarette (e-cigarette) use is increasing, cigarette use is decreasing and increasing numbers of adolescents report using both e-cigarettes and cigarettes. Several studies among US adolescents suggest that self-reported e-cigarette use is associated with subsequent initiation of cigarette use, whereas one study in US adolescents found no association between e-cigarette use and escalation of cigarette use. However, these studies were all conducted in the USA, did not validate their self-reported smoking measures against objective measures and assessed only a limited range of risk factors for smoking as covariates and moderators of these relationships.

**Interpretation:** Associations similar to those found in the previous studies are reported in a sample of UK adolescents and are validated against breath CO measures. Data collected over a 12-month period confirmed a sizeable relationship between ever use of e-cigarettes and subsequent initiation of cigarette use and showed that e-cigarette use is modestly associated with subsequent escalation of cigarette use. The former but not the latter relationship remained after controlling for various other risk factors for smoking (eg, intentions to smoke), only some of which had been assessed in previous studies. These findings support the robustness of the relationship between ever use of e-cigarettes and initiation of cigarette use but suggest the relationship between ever use of e-cigarettes and escalation of cigarette use may be explainable by other factors. Ever use of e-cigarettes was a stronger predictor of initiation of cigarette use in those with no friends who smoked at baseline compared with those with a few or most friends who smoked at baseline. The latter finding would not appear to be consistent with the suggestion that e-cigarette use may simply be a marker for those who would go on to smoke cigarettes even without having tried e-cigarettes.
